# Tourism Information Management System Using Neural Networks Driven by Particle Swarm Model

**DOI:** 10.1155/2022/6386360

**Published:** 2022-06-13

**Authors:** Xuan Gao, Yuan Qi, Yong Chai, Chun Lei, Jiefei Wang

**Affiliations:** ^1^School of International Hospitality Management, University of Sanya, Sanya 572022, China; ^2^Research Institution of Hainan Silk Road Commercial Civilization, University of Sanya, Sanya 572022, China; ^3^School of Tourism Management, University of Sanya, Sanya 572022, China; ^4^School of Hospitality, Tourism and Event, Taylor's University, Penang, Malaysia

## Abstract

Based on the concept of “smart tourism,” this paper designs and implements a tourism management information system based on PSO-optimized NN. The foreground tourism web page of the system adopts DIV + CSS mode for page planning and layout, PHP as the client script language, and SQL server as the database to store and analyze user information. At the same time, the system adds personalized components to the user's search ranking results, so that the routes and scenic spots presented in front of users in the result interface are more in line with users' consumption habits. In order to verify the performance of the model and algorithm constructed in this paper, several experiments were carried out in this paper. Experimental results show that the prediction accuracy of this algorithm is 94.67% and the recall rate is 96.11%. This algorithm can overcome the disadvantages of traditional algorithms and provide some effective suggestions for tourism management. At the same time, this paper applies the concept of “smart tourism” to specific tourism informatization, which can promote the transformation and upgrading of tourism industry structure and further enhance the overall development level of tourism industry.

## 1. Introduction

Tourism informatization contributes significantly to the growth of tourism and is increasingly becoming a key competitive advantage in global resource allocation [1]. The three major industries in the world are tourism, automobiles, and oil, and tourism is also known as the “smokeless industry.” The tourism industry has grown rapidly in tandem with the improvement of people's living standards, but it has also brought many problems. Travel agencies currently deal with a large amount of data, with low efficiency and significant errors. With the advancement of the economy and the improvement of people's living standards, many people are turning to holiday tourism to relieve stress and relax [2]. As a result, people's demand for tourism will continue to rise. It is critical to develop a tourism management information system that meets application requirements and has comprehensive functions in order to better adapt to the current market situation. Wisdom tourism is a higher-level and more comprehensive guiding strategy for tourism informatization development, as well as a more profound embodiment of the value of tourism informatization development. With the rapid advancement of computer technology, database management systems are becoming increasingly sophisticated [3]. People are gradually paying attention to modern enterprise management as a result of the establishment of scientific management systems and the popularisation of modern computer management mode, particularly due to the rapid development of computer technology and modern communication technology [4]. The use of computer-assisted management emerged and grew quickly. The importance of carrying out informatization construction in the tourism industry is both theoretical and practical.

People's office efficiency and cost savings can be greatly improved with a tourism management system. However, tourism development is currently slow, and there is no effective management strategy in place. Enterprises with poor information management are less efficient. It is not smooth due to a lack of informatization, a single management mode, and limited communication channels in the tourism administrative department. The tourism industry's development is hampered by these issues. Simultaneously, how to quickly and accurately find high-quality information from the vast amount of Internet data; how to thoroughly analyze customers' consumption habits and formulate user strategies is an important field of Internet technology research [5]. As an information processing system that mimics biological neurons, the ANN (Artificial Neural Network) offers non-linear approximation, parallel processing, self-learning, self-organization, and fault tolerance. As a result, it has some advantages in terms of practical application. The most widely used NN (Neural network) model is the Bpnn (Back propagation neural network) [6]. To adjust the weight, BPNN typically uses the gradient descent method. However, it has some flaws, such as the ability to fall into local minima, slow convergence speed, and easy oscillation [7]. This paper introduces PSO (Particle Swarm Optimization), which is used to train the weights and thresholds of NN in order to address these flaws. This paper builds a tourism management information system based on PSO-optimized NN based on the concept of “smart tourism.” The following are some of its innovations:

- This paper mainly researches and develops the system according to the idea of software engineering design, and uses a variety of cutting-edge technologies to complete the establishment of tourism management information system. Aiming at the time-consuming problem faced by users in choosing tourist routes, this paper puts forward a brand-new price comparison decision method. It can help users easily get all kinds of travel route information of various travel websites. In addition, the security and reliability of this system are effectively verified by testing.
- BPNN generally adopts gradient descent method to adjust weights. In this paper, an improved PSO-optimized NN algorithm is proposed, aiming at its inherent defects of falling into local minima, slow convergence and easy oscillation. Compared with the traditional NN algorithm, the results show that the performance of PSO-optimized NN algorithm is better.

This paper will be divided into five sections based on the content of the paper and the needs of the article structure, with the following contents for each section: the introduction is the first section. This section provides an overview of the paper's research background, significance, and innovation. The work in the second section is related. This section describes the current state of research on the research topic both at home and abroad, as well as the research work and ideas presented in this paper. There are two parts to the third section.  Section 3.1 examines the tourism management information system and the NN algorithm in general.  Section 3.2 A tourism management information system is built and implemented using a PSO-optimized NN algorithm. Many experiments are carried out in the fourth section to investigate the performance of the proposed system and the improved algorithm. Summary and prospects are covered in the fifth section. This chapter summarises the paper's research findings. Finally, the paper's shortcomings are discussed, as well as future research directions.

## 2. Related Work

With the fierce competition of more and more tourism industries, how to improve the service quality and management of tourism is becoming more and more important. At the same time, with the rapid development of mobile Internet, cloud computing, AI and other technologies, the integration of tourism industry and IT industry is getting closer and closer. among them, ANN has been well applied in many fields. At present, many scholars have combined NN with tourism and made some achievements.

In order to improve the prediction accuracy of popular tourist attractions, Du et al. proposed a prediction model of popular tourist attractions based on genetic algorithm optimization [8]. Zhou et al. used use cases for travel service recommendation [9]. This model organizes the user's historical data into case forms and stores them; when the user has a demand, the system will search through the case by the method of similarity calculation to find the case that is close to the user's needs; The case is adjusted according to local needs and a recommended solution is generated. Liu et al. introduced the radial basis function NN with strong non-linear modeling ability to describe the non-linear change characteristics of popular tourist attractions, and optimized the parameters of the radial basis function NN to establish the optimal prediction model of tourist attractions [10]. Bai and Han made dynamic prediction of tourist attractions based on grey system and NN [11]. Lv uses the improved genetic algorithm to optimize the structure and initial weight of the BPNN in the research of improving the genetic algorithm to optimize the BPNN for prediction [12]. In the PSO optimization NN prediction model, Zhao et al. used PSO to optimize the weight parameters of the BP network [13]. This method improves the problem that the BP network is easy to fall into the local minimum value, and improves the convergence speed. The system proposed by Sundriyal et al. uses demographic information to recommend tourist attractions, and classifies users by using Bayesian methods and support vector machines; and assumes that users in the same class have similar interests and preferences, recommending similar Other attractions of interest to users to solve the cold start problem [14]. It has a certain effect. Du achieves a balance between the global search and local search of particles through the linear decrease of the inertia weight and acceleration factor of the PSO, and improves the optimization performance [15]. Behjaty and Monfared proposed a PSO-optimized NN ensemble tourism prediction model based on support vector machines [16]. Zhang designed a tourism demand forecasting system based on BPNN. The system can dynamically predict tourism demand and has good prediction accuracy [17].

This paper discusses the mode of “smart tourism” and builds a tourism management information system based on PSO optimized NN based on an in-depth review of relevant literature. To begin, an improved PSO optimization algorithm is used to optimize the weight parameter combination of the BPNN model repeatedly. The algorithm is then used to precisely optimize the obtained network parameters. Finally, for prediction, the most accurate optimal parameter combination is used. Customer receipt management module, customer information management module, management module, customer group, tourism resource management module, and functional module have all been completed through design and practise. Finally, the experimental simulation demonstrates that the improved method presented in this paper improves the accuracy of prediction results while requiring few parameters and being simple and effective.

## 3. Methodology

### 3.1. Tourism Management Information System and NN Algorithm

**Figure 1:**
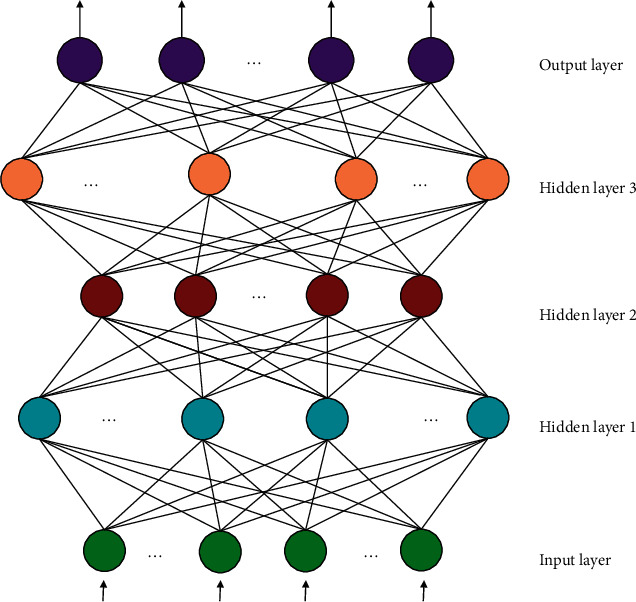
NN structure.

The brain is the most complex, perfect and effective information processing system known in the universe. The number of nerve cells in the brain is huge, and these nerve cells are also called neurons. It is hundreds of millions of such neurons that make up our human brain through complex connections. Neurons are the only cells in human body that can sense stimulation and conduct shock, and are also the basic units of nervous system in morphology, structure, function and nutrition. Inspired by the bionics research of ANN [18–20], the operation mode of brain neurons was simulated. NN has the characteristics of massive parallelism, learning autonomy, high nonlinearity and self-organization, and is widely used in many fields. ANN is a complex network composed of a large number of simple components connected to each other. It has a high degree of nonlinearity, and is a system that can perform complex logical operations and realize non-linear relationships. ANN can process information, and its core idea is to adjust the connection relationship between internal nodes according to the different complexity of the system. It has all the characteristics of non-linear dynamic system, such as irreversibility, various types of attractors, chaos and so on. BPNN is the most commonly used NN model at present. Because of its good adaptability and strong non-linear mapping ability, BPNN has been widely used, but it also has some shortcomings. For example: ① the random initialization assignment of BP network connection weight and threshold before network model training makes the network easy to fall into local extreme points and affects the accuracy of prediction. ② For the determination of BPNN structure, there is no exact formula for the number of hidden layer nodes. Improper selection is prone to over fitting or insufficient learning ability, which affects the generalization ability of the network. BPNN model generally has input layer, hidden layer and output layer. The neurons in adjacent layers are fully connected, and there is no connection between neurons in each layer. The structure of NN is shown in Figure 1.

BPNN is a multilayer feedforward NN with two processes: forward transmission of input signals and error back propagation. Various neurons in a feedforward network accept the previous layer's input and output to the next layer without receiving feedback. There are two types of nodes: input cells and calculation cells. There can be any number of inputs, but only one output per computing unit. The basic idea behind the BPNN algorithm is to first initialise the weight and threshold of NN, and then calculate the corresponding output value using the transfer function between NN layers; finally, calculate the error between the output value and the expected output value, and then reverse the weight and threshold of NN. NN is trained by performing forward and reverse error corrections repeatedly. The operation is terminated when the error value reaches the set standard. The neuron state of each layer only affects the neuron state of the next layer in the BPNN model. To approach any rational function with any accuracy, the network structure usually only requires a single hidden layer. The number of neural nodes in the input and output layers of the network is determined by the dimension of the input and output vectors of training samples. The characteristics of neurons, the form of neuron interconnection, and learning rules are the three factors that determine NN's overall performance. We must improve the BPNN model's inherent flaws in order to better apply it. PSO stands for swarm intelligence optimization. The algorithm is simple to use and is used in a variety of optimization problems. This paper will build a tourism management information system using a PSO optimized NN algorithm.

The development of information and Internet technology has completely changed people's life, and the intellectualization of tourism management has also become a trend. At present, the websites of travel agencies all over the country are in their own array in terms of function, information service and business operation, and the phenomenon of “information island” is serious [21]. The tourism management information system is convenient, which can manage, store and share resources through the network and various types of tourism resources. It greatly saves the labor cost, reduces the work difficulty and improves the intelligent level of the tourism industry. Continuously improve the level of tourism informatization management, optimize the allocation of resources, and realize the industrial chain of enterprises; It can promote the development of enterprises, expand employment and realize the positive role of tourism economic development. Therefore, developing a tourism management information system that can not only store but also update and query data is critical. The requirements for building various formats of informatization in smart tourism are extensive. The creation of a smart travel agency, for example, necessitates the network realization of various functions such as data collection and resource procurement, product planning and release, product sales, tourist service, order management, team management, statistical settlement, and so on. However, in the current reality, only a few tourism businesses use the network to carry out the aforementioned functions. The tourist route can be inquired by inputting tourist places; Travel agency management route; Recommended by popular scenic spots; Scenic spot reservation; Order processing, and so on are the main functions of a tourism management information system that can satisfy the application. It should also have a user-friendly interface and provide appropriate operation tips. Decision-making is central to management, and prediction [22, 23] is the foundation of decision-making. In tourism development and planning research, we should employ appropriate forecasting methods and models to determine the tourism economy's development law, as well as predict and speculate on tourism's future development trend, direction, and possibility, in order to better serve tourism planning, project decision-making, and management. Before developing an application system, we must understand the design and actual requirements of the system, assess the technology and principle's feasibility, and plan for development. The system should be simple to use once it has been launched, and most ordinary users with Internet experience should be able to use it without difficulty. At the same time, system administrators must have a thorough understanding of computers. General computer-related professionals can become competent after reading the instruction book. The paper then provides an overview of the system's overall design and implementation functions, as well as the system's overall test and trial operation, using an example design.

### 3.2. Construction of Tourism Management Information System Based on PSO-Optimized NN

**Figure 2:**
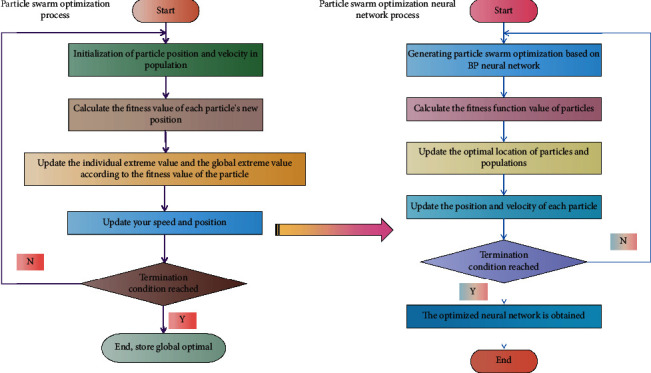
PSO optimization NN algorithm flow.

This system combines NN and PSO to create a tourism management information system. It is concerned with network applications. The function, or purpose, of a network management system should be the first step in its development [24]. The functional requirements are usually to clearly express a network management theme, to accommodate various types of content, and to adapt to various resolutions. Second, the design structure should be obvious. Because there are so many databases in this system, the databases must be designed ahead of time. It is the foundation of the data tourism management information system, the core content of the smart tourism city construction, and it provides crucial data support for the application service system construction. A data dictionary is made up of data items, data storage, data flow, data structure, and data processing, with data items being the smallest unit of the data dictionary. Data items make up the data structure. Data structure and data items are used to describe the data dictionary. A solution system for industry-wide data perception is proposed in this planning scheme, which is integrated into tourism intelligence data based on industry-wide data. Many weight adjustment methods for BPNN algorithms based on gradient descent exist, but they all have drawbacks, such as slow convergence, long training times, and global convergence. PSO's optimization process is independent of gradient information, and it does not require that the function be differentiable or that the derivative be excessive. PSO is used as the NN training method in this paper to reduce NN training time and improve search efficiency. The flow chart of optimizing PSO NN algorithm is shown in Figure 2.

A perfect system should have good stability, reliability, security and scalability, and can run efficiently. Therefore, this system meets the following performance requirements: ① correctness. ② Reliability. ③ Easy practicality. ④ Maintainability. ⑤ Reusability. ⑥ Understandability. ⑦ Safety. ⑧ Effectiveness. The system's basic information data for users primarily consists of gender, age, occupation, city, and historical comment items, while the comment data primarily consists of the user's comment text information on previous tourism service items and the corresponding scores. The system covers all end users based on the characteristics of the online travel network. The system also allows for real-time online operation, and the front page of timeliness and variability consistently sets high standards. The establishment of tourism data security standards ensures the security of data circulated in the tourism industry. Data transmission security standards, data storage security standards, and data exchange security standards are all included in the construction. Traditional linear modeling methods cannot accurately describe influencing factors and tourist attractions because they have a strong non-linear relationship. In order to fit the non-linear relationship between influencing factors and tourist attractions, this paper uses PSO to optimize NN. This paper uses a three-layer NN with only one hidden layer for the topological structure of NN in order to balance the algorithm's simulation and prediction accuracy, and because the more hidden layers, the more over-fitting of practical problems will occur. The number of nodes in the input layer represents the dimension of the original data, while the number of prediction indexes represents the number of nodes in the output layer. The activation function of a neuron and a network is what determines the network's function. Its primary function is to control the activation of input to output and to perform functional conversion on input and output in order to transform an infinite domain input into a limited range output. S-type function is used in this paper. The output formula of NN is

(1) $$\begin{array}{c} Y\left(X_{n}\right)=\sum_{i=1}^{I}W_{i}\Phi \left(X_{n},t_{i}\right). \end{array}$$

In the formula, *W*_*i*_ represents the weight between the nodes of the hidden layer and the output layer; *t*_*i*_ represents the center of the NN function; Φ() represents the NN function, which is defined as follows:

(2) $$\begin{array}{c} \Phi \left(x\right)=\exp\left(\frac{{\left\|x-c_{i}\right\|}^{2}}{\delta_{i}^{2}}\right), \end{array}$$

where *δ*_*i*_ represents the radius of the radial basis, and *c*_*i*_ represents the center of the response function. For the three-layer BPNN used in this paper, the optimization dimension is

(3) $$\begin{array}{c} D=m\times n+n\times k+n+k. \end{array}$$

Set the threshold vector from the input layer to the hidden layer as

(4) $$\begin{array}{c} A_{1}={\left[a_{11},a_{12},a_{13},\ldots ,a_{1n}\right]}^{T}. \end{array}$$

Set the threshold vector from hidden layer to output layer as

(5) $$\begin{array}{c} A_{2}={\left[a_{21},a_{22},a_{23},\ldots ,a_{2k}\right]}^{T}. \end{array}$$

Set the encoding on each particle dimension in the algorithm to

(6) $$\begin{array}{c} x=\left[IW_{11},\ldots ,IW_{mn}LV_{11},\ldots ,LV_{nk}a_{11},\ldots ,a_{1n}a_{21},\ldots ,a_{2k}\right]. \end{array}$$

The adjustment of weights and thresholds in BPNN is based on the mean square of errors. The fitness function optimized by PSO is used as the back-propagation function of the BP network error, and the equivalence relationship between the mean square value of the error and the fitness function of PSO is established. The objective function expression is

(7) $$\begin{array}{c} f_{i}=\frac{1}{N}\sum_{k=1}^{n}{\left[y\left(k\right)-y_{m}\left(k\right)\right]}^{2}. \end{array}$$

In the formula, *N* represents the total number of training samples, *f*_*i*_ represents the square error sum of the objective function; *y*(*k*) and *y*_*m*_(*k*) represent the target output value and actual output value of the objective function, respectively.

The related parameters are coded based on the setting of the NN structure, and the optimization dimension is represented by the weights and thresholds of the NN connection. Using cyclic iteration, the number of hidden layers is determined, and the optimal number and weight of NN hidden layers are found. The optimal position of the current particle and PSO is updated based on the fitness function value. Then, to generate a new group of PSO, update each particle's position and velocity. The individuals in the improved PSO optimization algorithm are coded into the weights and thresholds of the three-layer feedforward NN structure. Set the variable value intervals in the improved PSO optimization algorithm, then randomly initialise PSO's position and speed within the initialization range. The performance of particle local search and global search will decrease if the inertia factor of PSO is linearly adjusted, according to the basic PSO. The non-linear method is used to overcome the limitations of the linear decreasing method. At this time, the inertia factor expression is

(8) $$\begin{array}{c} w\left(k\right)=w_{\min}+\left(w_{\max}-w_{\min}\right)\exp\left(-\frac{25k}{T_{\max}}\right). \end{array}$$

For the problem of network evaluation, the widely used evaluation indicators include MSE (Mean squared error), RMSE (Root mean square error), MAE (Mean absolute error) and MAPE (Mean absolute percentage error). The specific expressions of these four indicators are given here:

(9) $$\begin{array}{c} \text{MSE}=\frac{1}{n}\sum_{k=1}^{n}{\left(y_{k}-{\overset{⌢}{y}}_{k}\right)}^{2},\\ \text{RMSE}=\sqrt{\frac{1}{n}\sum_{k=1}^{n}{\left(\left|y_{k}-{\overset{⌢}{y}}_{k}\right|\right)}^{2}},\\ \text{MAE}=\frac{1}{n}\sum_{k=1}^{n}\left|y_{k}-{\overset{⌢}{y}}_{k}\right|,\\ \text{MAPE}=\frac{1}{N}\sum_{k=1}^{n}\left|\frac{y_{k}-{\overset{⌢}{y}}_{k}}{y_{k}}\right|. \end{array}$$

Among them, *y*_*k*_ is the actual value, and ${\overset{⌢}{y}}_{k}$ is the output value.

Using the system's HTML page, manage and maintain the online travel network. The customer and logical interface are displayed using the presentation layer. The presentation layer represents users, and the presentation layer also represents the link between users and the system. The presentation layer realises the client's request, and the data is transmitted, primarily in the business layer, with the results displayed. The goal of the data exchange interface design is to create a system that is open, extensible, adaptable, efficient, and stable. The data exchange interface has its own hierarchical authority management mechanism, and it also manages the security authentication of data acquisition and storage for other data exchange application systems, as well as effectively encrypting the data during transmission. The goal of this paper is to promote online tourism, and the principle of serving users is used to design a tourism management system that meets the actual needs of tourism management. Furthermore, the goal of a simple and clear interface, stable functionality, low implementation cost, and meeting a variety of service tourism demands has been met. Provide assistance to tourism businesses in making decisions.

## 4. Result Analysis and Discussion

This system is developed in B/S mode, and the interface of presentation layer is realized through WEB browser, mainly for the realization of text, Flash, pictures and other functions. Through the client browser HTML form, URL and Flash request realization, the data is given to the programming model, and the system provides J2EE realization to develop and realize the system. In order to verify the practical application effect of the system, simulation experiments are carried out in this chapter.  Table 1 shows the parameter settings of simulation test environment.

**Figure 3:**
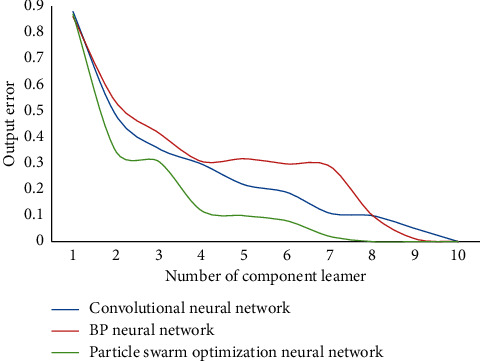
Training results of different networks.

Tansig function is used as the conversion function between the input layer and the hidden layer, and Purelin is used as the conversion function between the hidden layer and the output layer. The maximum training step is 1000, and the number of particles in the parasitic group and host group in the improved PSO is set to 50. The network is trained and the results are shown in Figure 3.

**Figure 4:**
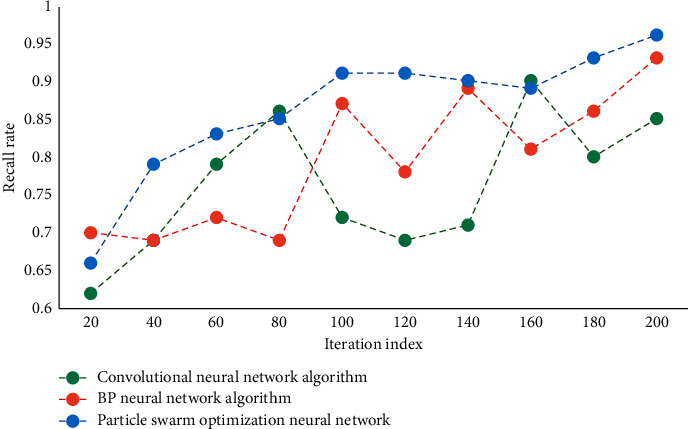
Comparison of recall rates of different algorithms.

**Figure 5:**
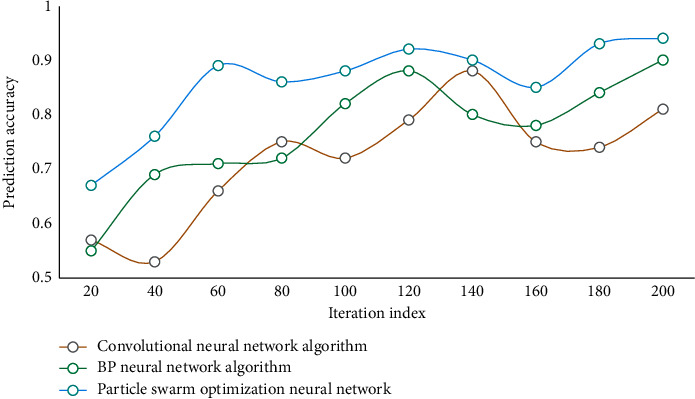
Comparison of prediction accuracy of different algorithms.

It can be seen that the convergence speed of this network is faster. In this paper, PSO is combined with NN, and the weight and threshold of NN are determined by using improved PSO instead of BPNN based on gradient descent. Therefore, the optimal parameter combination is obtained for NN to forecast the tourist volume in tourist areas. Three different models are used to predict the test set, and the prediction results are compared and the performance is analyzed. Test the recall results of different algorithms at the same time. The comparison results of recall rates of different algorithms are shown in Figure 4. The prediction accuracy results of different models are shown in Figure 5.

According to the data analysis in Figure 4, the recall rate of this algorithm is the highest, and it is better than the comparison algorithm. According to the data analysis in Figure 5, the prediction accuracy of this algorithm is at a high level, up to 94.67%. It shows that the model in this paper can be well applied to forecast the number of tourists in tourist areas. This further verifies the feasibility and effectiveness of this method.  Table 2 shows the test index values of different methods.

**Figure 6:**
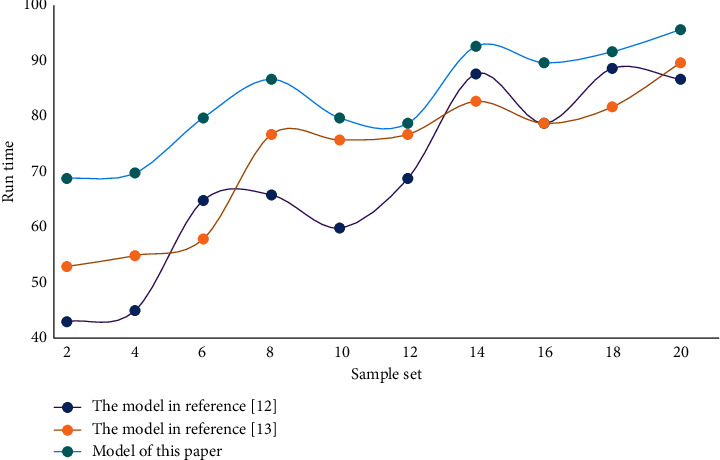
Running time results of different systems.

It can be seen from the data results in the table that the fitting accuracy of BPNN algorithm is much higher than that of cluster analysis algorithm; However, the accuracy of improved ANN is better than that of BPNN algorithm and traditional ANN. Comparing the performance of different systems, the running time results of different systems are shown in Figure 6.

**Figure 7:**
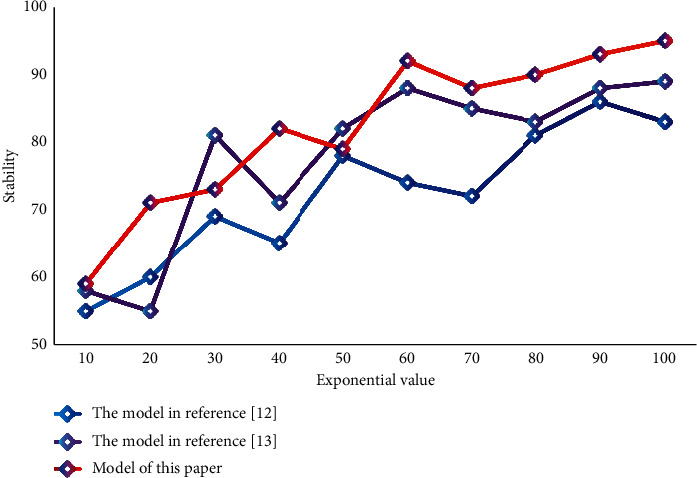
Stability test of different systems.

Considering the migration of different database management systems, SQLSERVER database replication is adopted. Through the data layer, it mainly operates the query, database storage, transaction processing and update of the system; Realize the return of database data results through the data layer. Test the stability of different systems as shown in Figure 7.

According to the data analysis in the figure, the stability of this system is higher than that of the other two comparison systems. It has the highest stability. Moreover, the response time of the system in this paper is short, which can achieve high efficiency. This data verifies that the system in this paper has certain superior performance. It can be applied to tourism information management. In this chapter, to verify the performance of the tourism management information system based on PSO-optimized NN proposed and designed in this paper, a simulation experiment is carried out. The simulation results show that the prediction accuracy of this algorithm is 94.67% and the recall rate is 96.11%. The performance of this system can meet the practical application requirements and provide some effective suggestions for tourism management decision-making.

## 5. Conclusions

The development of the tourism industry has been accelerated by the rapid development of computer technology, and the traditional tourism industry has changed dramatically. As a result, a tourism management information system with high availability, expandability, and ease of maintenance is required. This paper completes the construction and application of a tourism management information system using a PSO-optimized NN algorithm. The improved PSO is used to optimize BPNN in this paper, and the PSO's optimization performance is improved by nonlinearly decreasing the inertia factor. Simultaneously, the system provides end-to-end service, including system setup, management personnel analysis of tourism resource management, and statistical category reports. This can provide a great deal of convenience for visitors and have a significant positive impact on tourism benefits. Several experiments were conducted in order to verify the performance of the model and algorithm developed in this paper. The prediction accuracy of this algorithm is 94.67 percent, and the recall rate is 96.11 percent, according to experimental results. This system uses the Scrapy framework to crawl travel website route information, allowing it to efficiently and accurately capture travel route information; providing a user registration interface, clustering users based on user information, and storing user clicks in different categories on different lines; and rearranging route information to make search results more personalized and accurate. The system's performance is adequate for the application's needs. At the same time, this paper applies the concept of “smart tourism” to specific tourism informatization, which can help the tourism industry transform and upgrade its structure, as well as improve its overall development level. This research has made some achievements, but due to the time problem and the limitation of knowledge level, there are still many deficiencies in this system. In the future, distributed crawling and distributed storage should be considered to improve the system performance. At the same time, a more humanized interface should be designed.

## Figures and Tables

**Table 1:** Settings of simulation test environment parameters.

Serial number	Test parameters	Set up
1	Hidden layer	One
2	CPU	4 nuclear
3	RAM	64 GN
4	Hard disc	1T
5	Display card	512G
6	Operating system	Windows

**Table 2:** Test index values of different methods.

Algorithm	MSE	RMSE	MAE	MAPE
Convolution algorithm	0.154	0.499	0.803	0.614
BPNN algorithm	0.108	0.279	0.627	0.512
Traditional ANN algorithm	0.094	0.301	0.614	0.487
Improved PSO algorithm	0.057	0.213	0.551	0.369

## Data Availability

The data used to support the findings of this study are available from the corresponding author upon request.
